# The potential stickiness of pandemic-induced behavior changes in the United States

**DOI:** 10.1073/pnas.2106499118

**Published:** 2021-06-17

**Authors:** Deborah Salon, Matthew Wigginton Conway, Denise Capasso da Silva, Rishabh Singh Chauhan, Sybil Derrible, Abolfazl (Kouros) Mohammadian, Sara Khoeini, Nathan Parker, Laura Mirtich, Ali Shamshiripour, Ehsan Rahimi, Ram M. Pendyala

**Affiliations:** ^a^School of Geographical Sciences and Urban Planning, Arizona State University, Tempe, AZ 85281;; ^b^School of Sustainable Engineering and the Built Environment, Arizona State University, Tempe, AZ 85281;; ^c^Department of Civil, Materials, and Environmental Engineering, University of Illinois at Chicago, Chicago, IL 60607;; ^d^School of Sustainability, Arizona State University, Tempe, AZ 85281

**Keywords:** COVID-19, remote work, telecommuting, disruption, survey

## Abstract

Human behavior is notoriously difficult to change, but a disruption of the magnitude of the COVID-19 pandemic has the potential to bring about long-term behavioral changes. During the pandemic, people have been forced to experience new ways of interacting, working, learning, shopping, traveling, and eating meals. A critical question going forward is how these experiences have actually changed preferences and habits in ways that might persist after the pandemic ends. Many observers have suggested theories about what the future will bring, but concrete evidence has been lacking. We present evidence on how much US adults expect their own postpandemic choices to differ from their prepandemic lifestyles in the areas of telecommuting, restaurant patronage, air travel, online shopping, transit use, car commuting, uptake of walking and biking, and home location. The analysis is based on a nationally representative survey dataset collected between July and October 2020. Key findings include that the “new normal” will feature a doubling of telecommuting, reduced air travel, and improved quality of life for some.

Disruptions in our lives present opportunities to learn and practice new ways of doing things and to reevaluate old choices and habits (1). The COVID-19 pandemic has been perhaps the largest disruption event in modern human history. Nearly every human on the planet has been forced to modify their habits to adjust to the pandemic, creating an opportunity for long-term change. Importantly, the pandemic coincided in time with the widespread availability of technologies such as broadband internet service and videoconferencing, as well as many application-based services available through mobile phones.

To provide insights into the potential stickiness of pandemic-induced behavior changes, we developed an extensive survey and collected 7,613 responses in the United States between July and October 2020 (2). The dataset is weighted to be representative of US adults and captures prepandemic, pandemic-era, and expected future behavior in the areas of telecommuting, restaurant patronage, air travel, online shopping, transit use, car commuting, uptake of walking and biking, and home location.

We compare respondent expectations about their own future choices to their prepandemic lifestyles, contributing evidence-based estimates of how much pandemic-era changes may persist in the long run. We focus on expected changes that will be especially consequential for the US economy. Statistical modeling to ascertain the socioeconomic and geographical correlates of these changes is left for future work.

Although we recognize that stated intentions do not always accurately predict future choices, both the survey’s design and the choice context itself alleviate this concern. The survey instrument prompted respondents to provide reasons when they reported that they expect to behave differently postpandemic than was their prepandemic norm. These questions served both as a check on whether a change was actually expected and provided information that informs whether the change is likely to stick.

Further, respondents understand the choice context well. They experienced one lifestyle prepandemic, their daily lives changed during the pandemic, and our future-looking questions ask them how they plan to mix and match the two ways of life. Respondents have experience with both lifestyles as well as time to reflect on this question during the pandemic, so their answers are well-informed. One of our survey questions provides direct evidence of the “stickiness” of pandemic-induced behavior change; more than 70% of respondents indicated there were aspects of pandemic life they would like to continue.

Survey data documenting differences between prepandemic choices and expectations for the postpandemic future represent the direct, or partial equilibrium, effects of the pandemic. Substantial shifts in choices, however, will cause secondary effects to cascade through the economy, and government policies could shift as well. Estimating these secondary effects is beyond the scope of this brief report. We invite others to use this dataset (3) to calibrate general equilibrium models that can provide predictions of both primary and secondary effects.

## Telecommuting and Its Consequences

**Fig. 1.**
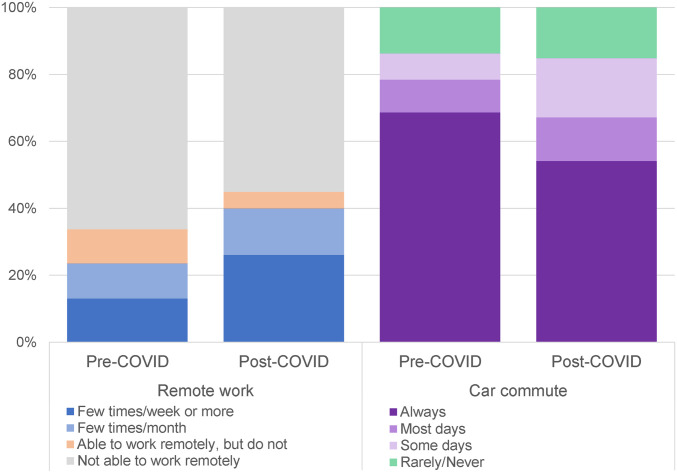
Key remote work shifts. Sample sizes: remote work = 4,554 employed adults; car commute = 3,217 commuters.

The most transformative long-term change identified in our data is a large increase in telecommuting. We asked respondents whether they expect to have the option to telecommute postpandemic and if so how often they expect to do so. Therefore, answers reflect individual preferences tempered by expectations about what their employers will allow. The fraction of workers who expect to telecommute at least a few times each week is double that of the prepandemic period, increasing from 13 to 26% (Fig. 1).

A shift to telecommuting is important for its direct impacts on quality of life, worker productivity, and commuting. Among those new to telecommuting at least a few times a week during the pandemic, two-thirds identified telecommuting and/or commuting less often as key features of pandemic life they would like to continue into the future. More than 70% of those new to regular telecommuting report that their productivity stayed the same or improved during the pandemic, consistent with prepandemic research (4). This is remarkable, since many pandemic-era telecommuters are juggling childcare and have suboptimal working environments.

The long-term increase in telecommuting is not equitably distributed across the population. Among workers who were not frequent telecommuters prepandemic, those who hold a bachelor’s degree or live in households earning over $100,000 per year are twice as likely to expect to telecommute at least a few times a week postpandemic. Thus, these quality-of-life improvements will flow primarily to high-income, highly educated individuals.

The direct impacts of telecommuting on car commuting are substantial. We estimate that less frequent commuting (Fig. 1) will reduce car commute kilometers by ∼15%. The fraction of commuters who choose the car as their primary commute mode is not expected to change substantially.

Telecommuting also impacts transit demand. Though transit systems carried just 5% of US commuters (5), commuting accounted for about half of all transit trips prepandemic (6). Our data suggest nearly a 40% decline in transit commute trips postpandemic, relative to prepandemic. Of this decline, about half can be attributed to changes in commuting frequency, 40% comes from a net shift among transit commuters toward the private car, and the remaining 10% comes from shifts to other modes.

A shift to telecommuting will have indirect effects on many aspects of our economy. There is likely to be reduced demand for office space and downtown parking. Patronage of office-district businesses is likely to decrease. Restaurants will continue to be hard-hit. Our data suggest that the number of people who plan to dine in restaurants at least a few times each week will decrease by more than 20% postpandemic, compared to the prepandemic era. Since the restaurant industry employed 8% of US workers prepandemic (7, 8), a decrease in restaurant patronage translates to a significant economic hardship for service workers.

## A Paradigm Shift in Air Travel

**Fig. 2.**
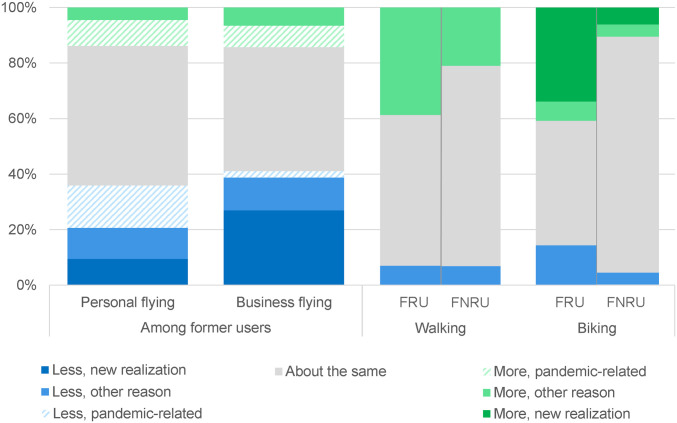
Prepandemic to postpandemic expected shifts in flying, walking, and biking, with reasons. A sizable fraction of survey respondents selected pandemic-related reasons for their expectations about their future choices. Full details are available in *SI Appendix*. FRU, former regular users; FNRU, former nonregular users. Sample sizes: personal flying = 5,313 former flyers; business flying = 1,676 former flyers; walking, FRU = 3,750; walking, FNRU = 3,794; biking, FRU = 1,000; biking, FNRU = 6,395.

Air travel demand dropped 95% at the height of the pandemic (9). Our data indicate that more than 40% of business travelers expect to travel less frequently postpandemic (Fig. 2). Of those reducing business travel, two-thirds attribute this change to new realizations that are likely to stick, primarily about the utility of videoconferencing. Personal air travelers also expect to fly less (Fig. 2), but nearly half of these reductions are caused by pandemic-related concerns that will likely soon fade.

## Accelerated Growth of Online Shopping for Groceries

The pandemic accelerated the uptake of online grocery shopping, nearly doubling the fraction of grocery spending done online (10). We analyzed survey responses from those who tried online grocery shopping for the first time during the pandemic. Approximately half expect to continue to grocery shop online at least a few times a month postpandemic, but nearly 90% of them also expect to shop in-store for groceries at least a few times a month. This suggests that online grocery shopping does not completely replace in-store shopping, although it may reduce its frequency. Among all US residents, 30% expect to grocery shop online at least a few times a month postpandemic, up from 21% prepandemic. Our data show online shopping for durable goods following a preexisting upward trend (11); 63% expect to shop for durable goods online at least a few times a month postpandemic, compared to 59% before the pandemic.

## Marked Increases in Walking and Bicycling

Biking and walking increased during the pandemic in many US cities (12), a change that improves both transport sustainability and public health. Postpandemic, 30% of US residents plan to take walks more frequently than they did before the pandemic, and nearly 15% plan to bike more (Fig. 2). These results include walking and biking for both transportation and recreation, with those who were frequent walkers or cyclists prepandemic expecting more change than those who were not. Overall, more than 20% identify taking more walks as one of the top three aspects of pandemic life they enjoy.

Many cities provided temporary infrastructure for walking and biking during the pandemic (13). To support a long-term shift, cities could make these changes permanent. Since commuting traffic is not expected to fully rebound, there is an opportunity to reallocate underutilized road space to pedestrians and bicyclists.

## Urban Exodus?

Some observers project a long-term decline of city centers as urbanites seek more space and no longer need to commute as often (14). Other research indicates the pandemic did not lead longtime urbanites to leave cities (15).

We compare reasons for moving between those who moved from dense urban neighborhoods and all other movers during the first 7 mo of the pandemic. The main difference was in the extent to which telecommuting opportunities motivated their moves. More than 20% of employed movers in dense urban areas cite not needing to commute as a reason for their move, as opposed to 9% of other employed movers. Likewise, 40% of employed movers in dense urban areas expect to telecommute at least a few times per week postpandemic, compared to 27% of other employed movers.

Notably, movers in dense urban areas were not more likely than other movers to be motivated by either pandemic-related public health concerns or by a desire for a more comfortable home.

The COVID Future dataset strongly suggests that society should expect and be planning for a “new normal.” Although only time will reveal the true impact of the pandemic, these data reflect our collective expectations of what the future will bring, providing important insights to help plan for what is next.

## Materials and Methods

The COVID Future survey dataset that is the basis for this article was collected between July and October 2020. The study protocol was approved by Institutional Review Boards at both Arizona State University and the University of Illinois at Chicago. Online consent was obtained from all survey respondents.

The data are weighted to represent the US population along the dimensions of gender, age, educational attainment, Hispanic status, income, vehicle ownership, and presence of children. All analysis presented here used these weights. A complete description of this dataset is available (2), and both the dataset and the survey questionnaire are available for download (3). *SI Appendix* provides details for all calculations.

## Supplementary Material

Supplementary File

Supplementary File

## Data Availability

Anonymized original survey data have been deposited in the ASU (Arizona State University) Dataverse (https://doi.org/10.48349/ASU/QO7BTC). All other study data are included in the article and/or supporting information.
