# Supplementary material for: The potential stickiness of pandemic-induced behavior changes in the United States
Source: Proc Natl Acad Sci U S A. 2021 Jun 17;118(27):e2106499118. doi: 10.1073/pnas.2106499118 (PMC8271792; doi:10.1073/pnas.2106499118)
Supplement: Supplementary File [file pnas.2106499118.sapp.pdf]

Supplementary Text for

The potential stickiness of pandemic-induced behavior changes in the United States

Deborah Salon, Matthew Wigginton Conway, Denise Capasso da Silva, Rishabh Singh Chauhan, Sybil Derrible, Kouros Mohammadian, Sara Khoeini, Nathan Parker, Laura Mirtich, Ali Shamshiripour, Ehsan Rahimi, and Ram Pendyala

This supplement provides details about how we analyzed the COVID Future survey data to arrive at the numbers that we cite in the article. It is written in Markdown so that it incorporates the Stata code used for analysis and can be both understood and replicated easily. All explanations are organized by section of the main text, and quotations from the main text are included so that it is clear which results are being documented.

Introduction

The results shared in this section of the article are directly calculated from simple tabulations of the survey data, as below.

- **"… more than 70% of respondents indicated there were aspects of pandemic life they would like to continue."**

The following tabulation illustrates that the exact percentage of the weighted sample that reported that they would definitely or maybe like to continue some aspect of pandemic life is 73.9%. In the survey, this question was followed by a list of possible pandemic-era lifestyle aspects, and respondents were instructed to select up to three of them. The top choices were "Working from home, at least some of the time", "Taking more walks", and "Spending more time with family".

```
. tab enjoychange_num [aw=weight_w1b]

      Enjoy |
     aspect |
   pandemic |      Freq.     Percent        Cum.
------------+-----------------------------------
         No | 1,987.1256       26.10       26.10
      Maybe | 3,393.2894       44.57       70.67
        Yes |  2,232.585       29.33      100.00
------------+-----------------------------------
      Total |      7,613      100.00
```

Remote work and its consequences

- **"The fraction of workers who expect to work remotely at least a few times each week is double that of the pre-pandemic period, increasing from 13% to 26%."**

The following tabulations illustrate. Note that these tabulations include only those workers who were employed pre-pandemic and expect to be employed post-pandemic.

```
. tab wfh_pre_comb3 if wfh_exp_comb3~=. [aw=weight_w1b]
```

Pre-pandemic remote work frequency	Freq.	Percent	Cum.
Unable	2,948.97721	66.21	66.21
Choose not to	455.476206	10.23	76.44
A few times/month	466.154541	10.47	86.90
More than once/week	583.392044	13.10	100.00
Total	4,454	100.00	

```
. tab wfh_exp_comb3 if wfh_pre_comb3~=. [aw=weight_w1b]
```

Expected post-pandemic remote work frequency	Freq.	Percent	Cum.
Unable	2,474.28919	55.55	55.55
Choose not to	216.891242	4.87	60.42
A few times/month	621.641647	13.96	74.38
More than once/week	1,141.1779	25.62	100.00
Total	4,454	100.00	

- **"Among those new to working remotely at least a few times a week, two-thirds identified remote work and/or commuting less often as key features of pandemic life they would like to continue into the future."**

The tabulations below indicate the fraction of those new to frequent remote work who value each of these aspects of pandemic life, and also the fraction who value either of them. Because we allowed survey respondents to choose up to three aspects of pandemic life that they value, many who were new to frequent remote work chose both of these. We identify those new to frequent remote work as workers who did not work remotely at least a few times a week pre-pandemic, and who do work remotely at least 2 times per week during the pandemic.

```
. tab enjoy_wfh_all [aw=weight_w1b] if wfh_now_days>1 & wfh_now_days~=. & wfh_pre
_comb3<3
```

Enjoy remote work	Freq.	Percent	Cum.
Not particularly	425.009273	39.72	39.72
Among my top 3 pandemic activities	644.990727	60.28	100.00
Total	1,070	100.00	

```
. tab enjoy_commute_less_all [aw=weight_w1b] if wfh_now_days>1 & wfh_now_days~=.
& wfh_pre_comb3<3
```

Enjoy commuting less	Freq.	Percent	Cum.
Not particularly	762.331253	71.25	71.25
Among my top 3 pandemic activities	307.668747	28.75	100.00
Total	1,070	100.00	

```
. tab enjoy_both [aw=weight_w1b] if wfh_now_days>1 & wfh_now_days~=. & wfh_pre_co
mb3<3
```

Enjoy remote work OR commuting less	Freq.	Percent	Cum.
Not particularly	350.406814	32.75	32.75
Among my top 3 pandemic activities	719.593186	67.25	100.00
Total	1,070	100.00	

- **"More than 70% of those new to regular remote work report that their productivity has stayed the same or improved during the pandemic."**

Note in the tabulation below that the "decreased" productivity categories added together sum to 27.7%. There is another category selected by 8.7% of those new to remote work: "in some ways it has increased and in other ways it has decreased". Because there are both effects for these workers, we included them in the "stayed the same" category in our reporting.

```
. tab prod_change [aw=weight_w1b] if wfh_now_days>1 & wfh_now_days~=. & wfh_pre_c
omb3<3
```

Change in work productivity	Freq.	Percent	Cum.
About the same	278.620595	26.04	26.04
Decreased significantly	50.7555587	4.74	30.78
Decreased somewhat	245.796212	22.97	53.75
In some ways it has increased and in ..	92.8835689	8.68	62.44
Increased significantly	107.7849548	10.07	72.51
Increased somewhat	294.159111	27.49	100.00
Total	1,070	100.00	

- **"Among workers who were not frequent telecommuters pre-pandemic, those who hold a bachelor's degree or live in households earning over $100,000 per year are twice as likely to expect to telecommute at least a few times a week post-pandemic."**

```
. tab freq_wfh_change bach [aw=weight_w1b], col nofreq
```

Expected post-pandemic change in WFH Frequency	bach No Bachel	Bachelor	Total
Stopped Freq WFH	1.52	2.56	1.92
Never Freq WFH	80.55	59.49	72.46
Always Freq WFH	8.31	15.77	11.18
Started Freq WFH	9.62	22.17	14.44
Total	100.00	100.00	100.00

```
. tab freq_wfh_change inc_over100K [aw=weight_w1b], col nofreq
```

Expected post-pandemic change in WFH Frequency	inc_over100K <$100K	>$100K	Total
Stopped Freq WFH	1.95	1.89	1.92
Never Freq WFH	78.84	64.85	72.46
Always Freq WFH	9.25	13.48	11.18
Started Freq WFH	9.97	19.78	14.44
Total	100.00	100.00	100.00

A limitation of the survey in the commuting section is important to mention. Those respondents who were not employed during the pandemic period were not asked what they expect their commute mode to be in the post-pandemic period. In addition, those who commuted for both employment and to school were only asked to provide details on the commute that was their primary activity. In all commuting analyses in this article, therefore, only those survey respondents who were employed in both the pre-pandemic period and the current period, and for whom work was their primary activity, are included.

- **"…we estimate that less frequent commuting will reduce car commute kilometers by approximately 15%."**

The total car commute distance decline is the net result of four effects:

1. Pre-pandemic car commuters expecting to switch away from cars.
2. Pre-pandemic non-car commuters expecting to switch to cars.
3. Pre-pandemic car commuters expecting to increase their remote work frequency without switching modes.

4. Pre-pandemic car commuters expecting to decrease their remote work frequency without switching modes.

To estimate the total change in car commute kilometers, we estimated car commute distances in both the pre- and post-pandemic periods for those survey respondents who were commuting to work in both periods, and calculated the percent change. To be sure that the change was largely due to less frequent commuting rather than mode switching, we also separately calculated the changes due to items (1), (2), and (3+4) above. The total percent change in car commute distance was 14.5%, which decomposed into a 15% decrease in car commute distance from changes in remote work frequency, and a 0.5% increase in car commute distance from mode switching.

To accomplish this calculation with the COVID Future survey data, however, a number of steps and some assumptions were required.

First, we needed to impute commute distances for the 285 pre-pandemic car commuters who did not provide them, but did provide commute times. To do this, we first calculated the average car commute speed for respondents who reported both car commute distances and times, and used this average speed to estimate commute distances for those car commuters who only reported commute times.

Calculating the average weekly car commute kilometers for private car commuters in the pre-pandemic period is straightforward: multiply the reported commute distance by the reported number of days per week of commuting.

For the post-pandemic period, the survey data do not include the number of days per week that each person expects to be commuting. The data do include both the pre-pandemic frequency of remote work and the expected post-pandemic frequency of remote work, however, which can be used together with the pre-pandemic number of days commuted per week to estimate post-pandemic commute days per week. Multiplying this by the reported commute distance gives us the post-pandemic expected weekly car commute kilometers.

Specifically, we estimate the post-pandemic commute frequency as follows.

1. If the pre- and post-pandemic frequency of remote work is the same, then we assume the post-pandemic commute frequency is the pre-pandemic number of days per week.
2. If the pre-pandemic frequency of remote work is “never” or “few times/year” and the post-pandemic frequency of remote work is “once/week” or “few times/month”, then we assume the post-pandemic commute frequency is the pre-pandemic number of days per week *minus one*.
3. If the pre-pandemic frequency of remote work is “never” or “few times/year” and the post-pandemic frequency of remote work is “few times/week”, then we assume the post-pandemic commute frequency is the pre-pandemic number of days per week *divided by two*.
4. If the pre-pandemic frequency of remote work is “once/week” or “few times/month” and the post-pandemic frequency of remote work is “few times/week”, then we assume

the post-pandemic commute frequency is the pre-pandemic number of days per week *plus one, divided by two*.

5. If the pre-pandemic frequency of remote work is “once/week” or “few times/month” and the post-pandemic frequency of remote work is “never” or “few times/year” then we assume the post-pandemic commute frequency is the pre-pandemic number of days per week *plus one*.
6. If the pre-pandemic frequency of remote work is “few times/week” and the post-pandemic frequency of remote work is “never” or “few times/year” then we assume the post-pandemic commute frequency is the pre-pandemic number of days per week *multiplied by two*.
7. If the pre-pandemic frequency of remote work is “few times/week” and the post-pandemic frequency of remote work is “once/week” or “few times/month”, then we assume the post-pandemic commute frequency is the pre-pandemic number of days per week *minus one, multiplied by two*.
8. If the post-pandemic frequency of remote work is “every day”, then we assume the post-pandemic commute frequency is *zero*.
9. If the reported post-pandemic primary mode choice is “I expect to work only from home and not commute”, then we assume the post-pandemic commute frequency is *zero*.
10. If the pre-pandemic reported frequency was *zero* because the person worked exclusively from home, but they expect to commute post-pandemic, then we add 5 commute days per week for those who expect not to work remotely, 4 commute days per week for those who expect to work remotely “once/week” or “few times/month”, and 2.5 commute days per week for those who expect to work remotely “few times/week”.
11. Finally, we adjust so that any resulting post-pandemic commute frequency that has been estimated to be less than zero is reset to zero, and any that has been estimated to be greater than seven is reset to seven. There are a small number of observations in each category.

The resulting average weekly car commute kilometers for the pre- and post-pandemic periods are illustrated below.

First, we calculate the total change in car commute distance for all of those who said that they commuted by car in either pre- or post-pandemic period AND who had non-missing numbers of commute days in both periods AND that had less than 500 car commute miles per week in both periods.

```
. quietly sum pre_car_commute_miles_week [aw=weight_w1b] if
    pre_work_com_days~=. &
    exp_work_com_days~=. &
    pre_work_pri_mode_w1b~="Question not displayed to respondent" &
    wcom_mode_exp_w1b~="Question not displayed to respondent" &
    wcom_mode_exp_w1b~="I do not expect to be employed in the future" &
    pre_car_commute_miles_week<500 &
    exp_car_commute_miles_week<500
```

```
. scalar pre_car_miles_total=r(sum)

. quietly sum exp_car_commute_miles_week [aw=weight_w1b] if
     pre_work_com_days~=. &
     exp_work_com_days~=. &
     pre_work_pri_mode_w1b~="Question not displayed to respondent" &
     wcom_mode_exp_w1b~="I do not expect to be employed in the future" &
     wcom_mode_exp_w1b~="Question not displayed to respondent" &
     pre_car_commute_miles_week<500 &
     exp_car_commute_miles_week<500

. scalar exp_car_miles_total=r(sum)

. scalar carmiles_change_total=pre_car_miles_total-exp_car_miles_total

. scalar pct_change_carmiles=(carmiles_change_total)/pre_car_miles_total

. display "Total percent change in car commute distance per week = " pct_change_c
armiles
Total percent change in car commute distance per week = .14544338
```

Next, we estimate the contributions to this net decrease in car commute distance that come from mode switching and changes in remote work frequency.

```
. *First, the contribution from those who shift away from cars
. quietly sum pre_car_commute_miles_week [aw=weight_w1b] if
     pre_work_pri_mode_w1b=="Private vehicle" &
     wcom_mode_exp_w1b~="Private vehicle" &
     wcom_mode_exp_w1b~="I expect to work only from home and not commute" &
     pre_work_com_days~=. &
     exp_work_com_days~=. &
     pre_work_pri_mode_w1b~="Question not displayed to respondent" &
     wcom_mode_exp_w1b~="Question not displayed to respondent" &
     wcom_mode_exp_w1b~="I do not expect to be employed in the future" &
     pre_car_commute_miles_week<500 &
     exp_car_commute_miles_week<500

. scalar carmiles_change_shift_away=r(sum)

. *Second, the contribution from those who shift to cars
. quietly sum exp_car_commute_miles_week [aw=weight_w1b] if
     pre_work_pri_mode_w1b~="Private vehicle" &
     wcom_mode_exp_w1b=="Private vehicle" &
     pre_work_com_days~=. &
     exp_work_com_days~=. &
     pre_work_pri_mode_w1b~="Question not displayed to respondent" &
     wcom_mode_exp_w1b~="Question not displayed to respondent" &
     wcom_mode_exp_w1b~="I do not expect to be employed in the future" &
     pre_car_commute_miles_week<500 &
     exp_car_commute_miles_week<500

. scalar carmiles_change_shift_to=r(sum)
```

```
. *Finally, the contribution from those pre-pandemic car commuters
. *who change their frequency of remote work
. quietly sum pre_car_commute_miles_week [aw=weight_w1b] if
    pre_work_pri_mode_w1b=="Private vehicle" &
    (wcom_mode_exp_w1b=="Private vehicle" |
    wcom_mode_exp_w1b=="I expect to work only from home and not commute") &
    pre_work_com_days~=. &
    exp_work_com_days~=. &
    pre_work_pri_mode_w1b~="Question not displayed to respondent" &
    wcom_mode_exp_w1b~="Question not displayed to respondent" &
    wcom_mode_exp_w1b~="I do not expect to be employed in the future" &
    pre_car_commute_miles_week<500 &
    exp_car_commute_miles_week<500

. scalar pre_carmiles_week3=r(sum)

. quietly sum exp_car_commute_miles_week [aw=weight_w1b] if
    pre_work_pri_mode_w1b=="Private vehicle" &
    (wcom_mode_exp_w1b=="Private vehicle" |
    wcom_mode_exp_w1b=="I expect to work only from home and not commute") &
    pre_work_com_days~=. &
    exp_work_com_days~=. &
    pre_work_pri_mode_w1b~="Question not displayed to respondent" &
    wcom_mode_exp_w1b~="Question not displayed to respondent" &
    wcom_mode_exp_w1b~="I do not expect to be employed in the future" &
    pre_car_commute_miles_week<500 &
    exp_car_commute_miles_week<500

. scalar exp_carmiles_week3=r(sum)

. scalar carmiles_change_wfh=pre_carmiles_week3-exp_carmiles_week3

. scalar pct_carmiles_change_wfh=carmiles_change_wfh/carmiles_change_total

. display "Fraction of change in car commute distance per week due to changes in
remote work = " pct_carmiles_change_wfh
Fraction of change in car commute distance per week due to changes in remote work
= 1.0302229
```

Importantly, in both this and the analysis of transit commute impacts of COVID, we assume that if people are working remotely on a given day, then they are not commuting on that day. Because the COVID Future survey is focused on impacts of the COVID-19 pandemic, we believe that this assumption is valid. A major impact of the pandemic has been that many workers have switched from working at a workplace to working from their homes. Therefore, the pandemic context clearly suggests that respondents to the COVID Future survey likely would interpret questions about remote work frequency to mean remote work *instead of* commuting to a workplace, rather than remote work *in addition to* commuting to a workplace.

In both analyses, we further assume that commuters use only their reported primary commute mode on the days when they travel to their workplace.

- **"The fraction of commuters who choose the car as their primary commute mode is not expected to change substantially."**

The following tabulations illustrate that 86% of U.S. workers were car commuters pre-pandemic, and this figure is expected to be 85% post-pandemic.

```
. tab pre_work_pri_mode_w1b [aw=weight_w1b] if
    pre_work_pri_mode_w1b~="Question not displayed to respondent" &
    wcom_mode_exp_w1b~="Question not displayed to respondent" &
    wcom_mode_exp_w1b~="I do not expect to be employed in the future"
```

Pre-pandemic primary commute mode	Freq.	Percent	Cum.
Other mode	39.3251632	1.22	1.22
Personal bicycle/scooter	33.7477706	1.05	2.27
Private vehicle	2,776.3097	86.30	88.57
Shared bicycle/scooter	14.962213	0.47	89.04
Transit	267.030076	8.30	97.34
Walk	85.6250465	2.66	100.00
Total	3,217	100.00	

```
. tab wcom_mode_exp_w1b [aw=weight_w1b] if
    wcom_mode_exp_w1b~="Question not displayed to respondent" &
    wcom_mode_exp_w1b~="I do not expect to be employed in the future" &
    pre_work_pri_mode_w1b~="Question not displayed to respondent"
```

Expected post-pandemic primary commute mode	Freq.	Percent	Cum.
I expect to work only from home and n..	117.837023	3.66	3.66
Other mode	22.2440893	0.69	4.35
Personal bicycle/scooter	49.936278	1.55	5.91
Private vehicle	2,730.1952	84.87	90.77
Shared bicycle/scooter	14.9091232	0.46	91.24
Transit	204.861871	6.37	97.61
Walk	77.0163873	2.39	100.00
Total	3,217	100.00	

- **"Commuting accounted for about half of all transit trips pre-pandemic."**

For comparison purposes, we determined the percentage of transit trips that were for commute purposes pre-COVID using the 2017 National Household Travel Survey. We subsetted the trips file to only transit trips (TRPTRANS codes 11 public/commuter bus, 12 paratransit, 15 Amtrak/commuter rail, and 16 Subway/elevated/light rail). We did not include boat trips as they may include both ferries and privately-operated boats, and excluded trips where the trip purpose was not included. We computed the weighted proportion of these trips which were for commuting to work or school purposes (either to work or from work).

- **"Our data suggest nearly a 40% decline in transit commute trips post-pandemic, relative to pre-pandemic."**

In order to arrive at this result, we use the assumptions outlined above to estimate the post-pandemic commute frequency per week. We then calculate the total number of commute trips per week by transit across all commuters for the pre- and post-pandemic periods, and compute the percent change.

```
. quietly sum pre_work_com_days [aw=weight_w1b] if
      pre_work_pri_mode_w1b=="Transit" &
      pre_work_com_days~=. &
      exp_work_com_days~=. &
      pre_work_pri_mode_w1b~="Question not displayed to respondent" &
      wcom_mode_exp_w1b~="I do not expect to be employed in the future" &
      wcom_mode_exp_w1b~="Question not displayed to respondent"

. scalar pre_trans_trips_week=r(sum)

. quietly sum exp_work_com_days [aw=weight_w1b] if
      wcom_mode_exp_w1b=="Transit" &
      pre_work_com_days~=. &
      exp_work_com_days~=. &
      pre_work_pri_mode_w1b~="Question not displayed to respondent" &
      wcom_mode_exp_w1b~="I do not expect to be employed in the future" &
      wcom_mode_exp_w1b~="Question not displayed to respondent"

. scalar exp_trans_trips_week=r(sum)

. scalar trans_change_total=pre_trans_trips_week-exp_trans_trips_week

. scalar pct_change_transit=(trans_change_total)/pre_trans_trips_week

. display "Total percent change in transit commute days per week = " pct_change_t
ransit
Total percent change in transit commute days per week = .38953326
```

- **"About half of this decline can be attributed to changes in the frequency of remote work, while the remainder comes from commute mode shifts."**

The analysis behind this statement is exactly analogous to that which we conducted for car commute distance. For transit, we do not calculate the change in distance commuted by transit but instead focus only on the change in the number of transit trips.

The total transit commute trip decline is the net result of four effects:

1. Pre-pandemic transit commuters expecting to switch away from transit.
2. Pre-pandemic non-transit commuters expecting to switch to transit.
3. Pre-pandemic transit commuters expecting to increase their remote work frequency without switching modes.

4. Pre-pandemic transit commuters expecting to decrease their remote work frequency without switching modes.

Here, we estimate the portion of the total transit commute trip decline that is due to the last two of these. For completeness, the following Stata code calculates the transit commute trip changes from expected mode shifts as well as that from changes in the frequency of remote work. Together these changes equal the total change in transit demand.

```
. *First, the contribution from those who shift away from transit
. quietly sum pre_work_com_days [aw=weight_w1b] if
     pre_work_pri_mode_w1b=="Transit" &
     wcom_mode_exp_w1b~="Transit" &
     wcom_mode_exp_w1b~="I expect to work only from home and not commute" &
     pre_work_com_days~=. &
     exp_work_com_days~=. &
     pre_work_pri_mode_w1b~="Question not displayed to respondent" &
     wcom_mode_exp_w1b~="I do not expect to be employed in the future" &
     wcom_mode_exp_w1b~="Question not displayed to respondent"

. scalar trans_trips_change_shift_away=r(sum)

. *Second, the contribution from those who shift to transit
. quietly sum exp_work_com_days [aw=weight_w1b] if
     pre_work_pri_mode_w1b~="Transit" &
     wcom_mode_exp_w1b=="Transit" &
     pre_work_com_days~=. &
     exp_work_com_days~=. &
     pre_work_pri_mode_w1b~="Question not displayed to respondent" &
     wcom_mode_exp_w1b~="I do not expect to be employed in the future" &
     wcom_mode_exp_w1b~="Question not displayed to respondent"

. scalar trans_trips_change_shift_to=r(sum)

. *Third, the contribution from changes in remote work frequency
. *This group are transit users in both periods, so calculate both periods
. *and take the difference
. quietly sum pre_work_com_days [aw=weight_w1b] if
     pre_work_pri_mode_w1b=="Transit" &
     (wcom_mode_exp_w1b=="Transit" |
     wcom_mode_exp_w1b=="I expect to work only from home and not commute") &
     pre_work_com_days~=. &
     exp_work_com_days~=. &
     pre_work_pri_mode_w1b~="Question not displayed to respondent" &
     wcom_mode_exp_w1b~="I do not expect to be employed in the future" &
     wcom_mode_exp_w1b~="Question not displayed to respondent"

. scalar pre_trans_trips_week3=r(sum)

. quietly sum exp_work_com_days [aw=weight_w1b] if
     pre_work_pri_mode_w1b=="Transit" &
     (wcom_mode_exp_w1b=="Transit" |
     wcom_mode_exp_w1b=="I expect to work only from home and not commute") &
```

```
    pre_work_com_days~=. &
    exp_work_com_days~=. &
    pre_work_pri_mode_w1b~="Question not displayed to respondent" &
    wcom_mode_exp_w1b~="I do not expect to be employed in the future" &
    wcom_mode_exp_w1b~="Question not displayed to respondent"

. scalar exp_trans_trips_week3=r(sum)

. scalar trans_trips_change_wfh=pre_trans_trips_week3-exp_trans_trips_week3

. scalar pct_trans_change_wfh=trans_trips_change_wfh/trans_change_total

. display "Fraction of change in transit commute days per week due to changes in
remote work = " pct_trans_change_wfh
Fraction of change in transit commute days per week due to changes in remote work
= .48711312
```

- **"Of this decline, about half can be attributed to changes in commuting frequency, 40% comes from a net shift among transit commuters toward the private car, and the remaining 10% comes from shifts to other modes."**

The first part of this sentence is calculated above at 48.7%. To calculate the fraction that comes from a net shift toward the private car versus other modes, the following additional code is required.

```
. quietly sum pre_work_com_days [aw=weight_w1b] if
    pre_work_pri_mode_w1b=="Transit" &
    wcom_mode_exp_w1b=="Private vehicle" &
    wcom_mode_exp_w1b~="I expect to work only from home and not commute" &
    pre_work_com_days~=. &
    exp_work_com_days~=. &
    pre_work_pri_mode_w1b~="Question not displayed to respondent" &
    wcom_mode_exp_w1b~="I do not expect to be employed in the future" &
    wcom_mode_exp_w1b~="Question not displayed to respondent"

. scalar pretrans_to_car=r(sum)

. quietly sum exp_work_com_days [aw=weight_w1b] if
    pre_work_pri_mode_w1b=="Private vehicle" &
    wcom_mode_exp_w1b=="Transit" &
    pre_work_com_days~=. &
    exp_work_com_days~=. &
    pre_work_pri_mode_w1b~="Question not displayed to respondent" &
    wcom_mode_exp_w1b~="I do not expect to be employed in the future" &
    wcom_mode_exp_w1b~="Question not displayed to respondent"

. scalar precar_to_trans=r(sum)

. scalar pct_tofrom_car=(pretrans_to_car - precar_to_trans)/trans_change_total
```

```
. disp "Percent change in transit commutes/week from shift to/from private car
 = " round(pct_tofrom_car*100) "%"
Percent change in transit commutes/week from shift to/from private car = 41%
```

- **"Our data suggest that the number of people who plan to dine in restaurants at least a few times each week will decrease by more than 20% post-pandemic, compared to the pre-pandemic era."**

This result is derived from the data in the following tabulations.

```
. tab shdi_pre_4 [aw=weight_w1b]
```

Pre-pandemic restaurant dining frequency	Freq.	Percent	Cum.
Never	352.603441	4.63	4.63
A few times/year	1,185.9179	15.58	20.21
A few times/month	3,628.6267	47.66	67.87
A few times/week	2,321.8066	30.50	98.37
Every day	124.04527	1.63	100.00
Total	7,613	100.00	

```
. tab shdi_exp_restaurant_dinein_w1b [aw=weight_w1b]
```

Expected post-pandemic restaurant dining frequency	Freq.	Percent	Cum.
Never	422.711151	5.55	5.55
A few times/year	1,505.2189	19.77	25.32
A few times/month	3,761.4631	49.41	74.73
A few times/week	1,791.3902	23.53	98.26
Every day	132.216646	1.74	100.00
Total	7,613	100.00	

Before the pandemic = 2,321.81+ 124.04= 2,445.85

After the pandemic = 1,791.39 + 132.22= 1,923.61

Ratio = 1,923.61/2,445.85 = 0.7867

- **"…the restaurant industry alone employed 8% of American workers pre-pandemic…"**

This figure is calculated by dividing the employment in the "food services and drinking places" industry (12.308 million) by total non-farm employment in the U.S. (152.523 million) for February 2020.

Figure 1

Figure 1 illustrates how the pandemic is expected to change demand for remote work and car commuting. The data behind this chart come from the following tabulations. Car commuting frequency here is calculated more simply than described above. Specifically, those who “Always” car commute report a private vehicle as their primary commute mode and that they work remotely “Never” or only “Few times/year”. Those who car commute “Most days” are car commuters who work remotely “Few times/month” or “Once/week”. Those who car commute “Some days” are car commuters who work remotely “Few times/week”. Finally, those who car commute “Never” either work remotely “Every day” or commute using another transport mode.

As explained above, the commuting analysis is limited to those who answered the survey questions about commute mode for both the pre-pandemic and post-pandemic periods.

```
. tab wfh_pre_comb3 [aw=weight_w1b]
```

Pre-pandemic remote work frequency	Freq.	Percent	Cum.
Unable	2,948.97721	66.21	66.21
Choose not to	455.476206	10.23	76.44
A few times/month	466.154541	10.47	86.90
More than once/week	583.392044	13.10	100.00
Total	4,454	100.00	

```
. tab wfh_exp_comb3 [aw=weight_w1b]
```

Expected post-pandemic remote work frequency	Freq.	Percent	Cum.
Unable	2,497.3628	55.03	55.03
Choose not to	226.099571	4.98	60.01
A few times/month	628.921281	13.86	73.87
More than once/week	1,185.6163	26.13	100.00
Total	4,538	100.00	

```
. tab pre_car_comm_freq [aw=weight_w1b] if exp_car_comm_freq~=.
```

Pre-pandemic car commute frequency	Freq.	Percent	Cum.
Always	2,210.0229	68.70	68.70
Most days	313.525703	9.75	78.44
Some days	252.761104	7.86	86.30
Never	440.690269	13.70	100.00
Total	3,217	100.00	

```
. tab exp_car_comm_freq [aw=weight_w1b] if pre_car_comm_freq~=.
```

Expected post-pandemic car commute frequency	Freq.	Percent	Cum.
Always	1,743.6142	54.20	54.20
Most days	417.409609	12.98	67.18
Some days	569.171418	17.69	84.87
Never	486.804772	15.13	100.00
Total	3,217	100.00	

Figure 2

Figure 2 illustrates how the pandemic is expected to change demand for both air travel and non-motorized mode use. The survey questions that form the basis for the figure included questions about the frequency of engaging in these activities pre-pandemic, how that frequency will change post-pandemic, and, for changes in expected air travel and bicycling frequency, we asked why. Specifically, the survey questions were:

- "How much do you expect your airplane travel for leisure/personal (business) purposes to change once COVID-19 is no longer a threat, compared to your level of travel before the COVID-19 pandemic?"
- "Why do you anticipate an increase/decrease in your long-distance travel for leisure/personal (business) purposes after COVID-19 is no longer a threat? Select all that apply." (separate questions for increase and decrease, depending on the person's actual response to the previous question)
- "After COVID-19 is no longer a threat, how do you expect your use of the following means of transport to change, relative to before the COVID-19 pandemic?" (The prompt also included the text "Please include any walks or bike rides for exercise or enjoyment.")
- "Why do you expect to increase your use of bicycles? Please select all that apply."

The air travel portion of the Figure focuses only on those who had traveled by airplane at least once per year pre-pandemic for leisure/personal and, separately, for business purposes (based on a separate question about pre-pandemic air travel). Those who expect to increase/decrease air travel for each purpose were then asked to select the reasons why, which we separated into "Pandemic-related", "New realization", and "Other" categories, as follows:

- Leisure Only, Less, new realization: "I am able to use technology (e.g., FaceTime, Zoom) to meaningfully engage with long-distance connections"
- Business Only, Less, new realization: "I realized I could conduct my meetings by conference call/video conference"
- Business Only, Less, new realization: "Those I meet with have realized that we can conduct meetings by conference call/video conference"

- Leisure and Business, Less, new realization: “I want to spend more time at home”
- Leisure and Business, Less, other reason: “I anticipate taking more of my long-distance trips by car”
- Leisure and Business, Less, other reason: “I anticipate taking more of my long-distance trips by train or bus”
- Leisure and Business, Less, other reason: “I want to fly less for environmental reasons”
- Business Only, Less, other reason: “My employer adopted a commitment to reduce travel by airplane”
- Business Only, Less, other reason: “My job responsibilities have changed”
- Business Only, Less, other reason: “I expect reduced budget for travel”
- Leisure and Business, Less, other reason: “Other, please specify”
- Leisure and Business, Less, pandemic-related: “I will not feel safe or comfortable sharing close space with strangers”
- Leisure Only, Less, pandemic-related: “My financial circumstances changed and I can no longer afford to travel in the same way”
- Leisure and Business, More, pandemic-related: “I will need/want to take trips that were cancelled during the COVID-19 pandemic”
- Leisure Only, More, pandemic-related: “After having been cooped up at home for so long, I want to travel more than I did before”
- Leisure Only, More, other reason: “My financial circumstances changed and I can now afford more air travel”
- Business Only, More, other reason: “My job responsibilities have changed”
- Leisure and Business, More, other reason: “Other, please specify”

If a respondent selected both a “New realization” reason and an “Other” reason, their response was categorized as a “New realization” response. If a respondent selected both an “Other” reason and a “Pandemic-related” reason, their response was categorized as an “Other” response. Therefore, those categorized as “Pandemic-related” are those who *only* selected a “Pandemic-related” reason.

With these definitions, below are the tabulations of the proportion of the sample in each category for personal and business air travelers that are illustrated in Figure 2.

```
. tab ld_bz_exp_reason [aw=weight_w1b]
```

Expected post-pandemic business air travel w/reasons	Freq.	Percent	Cum.
Less, new realization	452.464562	27.01	27.01
Less, other reason	197.081616	11.77	38.78
Less, pandemic-related	38.2287998	2.28	41.06
About the same	749.003526	44.72	85.78
More, pandemic-related	129.11137	7.71	93.49
More, other reason	109.110126	6.51	100.00
Total	1,675	100.00	

```
. tab ld_per_exp_reason [aw=weight_w1b]
```

Expected post-pandemic personal air travel w/reasons	Freq.	Percent	Cum.
Less, new realization	413.306654	7.78	7.78
Less, other reason	641.044088	12.07	19.84
Less, pandemic-related	856.78342	16.13	35.97
About the same	2,666.7692	50.19	86.16
More, pandemic-related	497.236891	9.36	95.52
More, other reason	237.859707	4.48	100.00
Total	5,313	100.00	

The walking portion of Figure 2 is straightforward, since there was not a survey question that asked about reasons for expected increases or decreases in walking frequency post-pandemic. Below is the tabulation of the proportion of the sample in each category for walking. Figure 2 puts the “Somewhat” and “Much” categories together for both “More” and “Less” walking.

```
. tab tr_freq_exp_walk_w1b [aw=weight_w1b] if tr_freq_exp_walk_w1b~="Seen but una
nswered"
```

Expected post-pandemic walking frequency	Freq.	Percent	Cum.
About the same	4,803.8198	63.53	63.53
Much less than before	253.149255	3.35	66.87
Much more than before	707.520755	9.36	76.23
Somewhat less than before	280.23573	3.71	79.94
Somewhat more than before	1,517.2744	20.06	100.00
Total	7,562	100.00	

Figure 2 also separates those who were Former Regular Users (FRU) of walking from those who were not, with a regular user defined as someone who walked a few times a week or more pre-pandemic.

```
. tab tr_freq_exp_walk_w1b [aw=weight_w1b] if tr_freq_exp_walk_w1b~="Seen but una
nswered" & (tr_freq_pre_walk_w1b==3 | tr_freq_pre_walk_w1b==4)
```

Expected post-pandemic walking frequency	Freq.	Percent	Cum.
About the same	2,037.98047	54.35	54.35
Much less than before	121.013992	3.23	57.57
Much more than before	527.477842	14.07	71.64
Somewhat less than before	142.198825	3.79	75.43
Somewhat more than before	921.32887	24.57	100.00
Total	3,750	100.00	

```
. tab tr_freq_exp_walk_w1b [aw=weight_w1b] if tr_freq_exp_walk_w1b~="Seen but una
nswered" & tr_freq_pre_walk_w1b<3
```

Expected post-pandemic walking frequency	Freq.	Percent	Cum.
About the same	2,740.7571	72.24	72.24
Much less than before	125.372119	3.30	75.54
Much more than before	188.0367924	4.96	80.50
Somewhat less than before	137.971133	3.64	84.14
Somewhat more than before	601.862902	15.86	100.00
Total	3,794	100.00	

Calculating the biking portion of Figure 2 was complicated by the fact that the survey asked respondents separately about biking using personal bicycles and biking using shared bicycles, and this figure combines the two. To accomplish this, we assume the following:

- a person expects to bike less post-pandemic than they did pre-pandemic if they expect to use either personal or shared bicycles “much less”, and they do not expect to use the other bicycle type “much more”
- a person expects to bike less post-pandemic than they did pre-pandemic if they expect to use either personal or shared bicycles “somewhat less”, and they do not expect to use the other bicycle type “somewhat more” or “much more”
- a person expects no change in their biking if they expect to bike “about the same” in both bicycle types
- a person expects no change in their biking if they expect to bike “somewhat less” for one bicycle type and “somewhat more” for the other, or “much less” for one and “much more” for the other
- a person expects to bike more post-pandemic than they did pre-pandemic if they expect to use either personal or shared bicycles “much more”, and they do not expect to use the other bicycle type “much less”

- a person expects to bike more post-pandemic than they did pre-pandemic if they expect to use either personal or shared bicycles “somewhat more”, and they do not expect to use the other bicycle type “somewhat less” or “much less”

Figure 2 also includes reasons for expected increases in biking, but not reasons for expected decreases. The reasons for expected increases are categorized into “New realization” and “Other” reasons, as follows:

- More, new realization: “I realized I really like biking”
- More, new realization: “I realized biking is fast”
- More, new realization: “I bought a bike”
- More, new realization: “I realized biking is an inexpensive way to get around”
- More, other reason: “I expect my city to make biking safer”
- More, other reason: “I expect to bike more in my neighborhood”
- More, other reason: “I expect to use biking to replace trips by other means of transport”
- More, other reason: “Other, please specify”

If a respondent selected both a “New realization” reason and an “Other” reason, their response was categorized as a “New realization” response.

Finally, Figure 2 illustrates differences in walking and biking expectations for Former Regular Users (FRU) and Former Non-Regular Users (FNRU). As with walking, these are defined as people who used each mode pre-pandemic a few times a week or more. Again, this is somewhat complicated for biking because the survey asked respondents about frequency of bike use separately for personal and shared bicycles. Here, we assume that a person is a FRU if they used either type of bicycle a few times a week or more pre-pandemic, or if they used both types of bicycle a few times a month pre-pandemic.

With these definitions, below are the tabulations of the proportion of the sample overall and in each pre-pandemic user frequency category for biking that are illustrated in Figure 2.

```
. tab bike_exp_reason [aw=weight_w1b]
```

Expected biking frequency w/reasons	Freq.	Percent	Cum.
Less	438.510214	5.93	5.93
About the same	5,856.7007	79.20	85.13
More, other reason	346.3236583	4.68	89.81
More, new realization	753.465462	10.19	100.00
Total	7,395	100.00	

```
. tab bike_exp_reason [aw=weight_w1b] if bike_pre_high==0
```

Expected biking frequency w/reasons	Freq.	Percent	Cum.
Less	286.6207111	4.48	4.48
About the same	5,441.5458	85.09	89.57
More, other reason	274.027911	4.29	93.86
More, new realization	392.805625	6.14	100.00
Total	6,395	100.00	

```
. tab bike_exp_reason [aw=weight_w1b] if bike_pre_high==1
```

Expected biking frequency w/reasons	Freq.	Percent	Cum.
Less	144.090574	14.41	14.41
About the same	446.8948388	44.69	59.10
More, other reason	70.1509578	7.02	66.11
More, new realization	338.863629	33.89	100.00
Total	1,000	100.00	

A paradigm shift in air travel

All of the specific numbers in this section of the article can be derived from the air travel-related information that is represented in Figure 2. Please see Figure 2's explanation for details.

Accelerated growth of online shopping for groceries

We identified those who were new to online grocery shopping as people who reported that they "Never" shopped online for grocery delivery or pickup at the store pre-pandemic, and that they did one or both of these activities within the seven-day period before taking the survey during the pandemic. There are undoubtedly others in this sample that also tried online grocery shopping during the pandemic, but did not happen to do so during the week before taking this survey. That said, the subsample that we have identified here as new to online grocery shopping is 780 people - large enough to draw conclusions from.

The remaining results reported in this section of the article are directly calculated from simple tabulations of the survey data, as below.

- **"We analyzed survey responses from those who tried online grocery shopping for the first time during the pandemic. Approximately half expect to continue to grocery shop online at least a few times a month post-pandemic..."**

```
. tab exp_groc_online [aw=weight_w1b] if new_groc_online==1
```

Expected post-pandemic online grocery shop frequency	Freq.	Percent	Cum.
0	398.346704	51.07	51.07
1	381.653296	48.93	100.00
Total	780	100.00	

- **"… nearly 90% of them also expect to shop in-store for groceries at least a few times a month."**

```
. tab shdi_exp_groceries_instore_w1b [aw=weight_w1b] if new_groc_online==1
```

Expected post-pandemic in-store grocery shop	Freq.	Percent	Cum.
Never	23.8746581	3.06	3.06
A few times/year	76.1217172	9.76	12.82
A few times/month	382.186539	49.00	61.82
A few times/week	282.392375	36.20	98.02
Every day	15.4247104	1.98	100.00
Total	780	100.00	

- **"This suggests that online grocery shopping does not completely replace in-store shopping, although it may reduce its frequency."**

We investigated whether those new to online grocery shopping also expect to reduce their frequency of in-store shopping, and found that many of them do. Specifically, among respondents who were new to online shopping during the pandemic and also reported that they would still be shopping in person at least a few times each month post-pandemic, 25% of them report that they expect to shop in grocery stores less frequently. This compares to about 10% of respondents who did not try online grocery shopping for the first time during the pandemic. See below for the code that provides these numbers.

```
. tab instore_groc_decr [aw=weight_w1b] if new_groc_online==1 &
instore_pre_groc_cond~=1 & instore_exp_groc_cond~=1
```

Expected decrease in in-store grocery shop frequency	Freq.	Percent	Cum.
0	506.632054	74.72	74.72
1	171.367946	25.28	100.00
Total	678	100.00	

```
. tab instore_groc_decr [aw=weight_w1b] if new_groc_online==0 &
instore_pre_groc_cond~=1 & instore_exp_groc_cond~=1
```

Expected decrease in in-store grocery shop frequency	Freq.	Percent	Cum.
0	5,605.9738	88.55	88.55
1	725.026185	11.45	100.00
Total	6,331	100.00	

- **"Among all U.S. residents, 30% expect to grocery shop online at least a few times a month post-pandemic, up from 21% pre-pandemic."**

```
. tab exp_groc_online [aw=weight_w1b]
```

Expected post-pandemic online grocery shop frequency	Freq.	Percent	Cum.
0	5,366.0266	70.49	70.49
1	2,246.9734	29.51	100.00
Total	7,613	100.00	

```
. tab pre_groc_online [aw=weight_w1b]
```

Pre-pandemic online grocery shop frequency	Freq.	Percent	Cum.
0	6,023.0379	79.12	79.12
1	1,589.9621	20.88	100.00
Total	7,613	100.00	

- **“63% of people expect to shop for durable goods online at least a few times a month post-pandemic, compared to 59% before the pandemic.”**

```
. tab shdi_pre_7 [aw=weight_w1b]
```

Pre-pandemic online non-grocery shop	Freq.	Percent	Cum.
Never	1,304.4542	17.13	17.13
A few times/year	1,848.6911	24.28	41.42
A few times/month	3,117.4766	40.95	82.37
A few times/week	1,211.4052	15.91	98.28
Every day	130.972874	1.72	100.00
Total	7,613	100.00	

```
. tab shdi_exp_onlineother_w1b [aw=weight_w1b]
```

Expected post-pandemic online non-grocery shop	Freq.	Percent	Cum.
Never	1,151.4118	15.12	15.12
A few times/year	1,632.4737	21.44	36.57
A few times/month	3,235.0604	42.49	79.06
A few times/week	1,432.4196	18.82	97.88
Every day	161.63451	2.12	100.00
Total	7,613	100.00	

Marked increases in walking and bicycling

The results reported in this section of the article are directly calculated from simple tabulations of the survey data, as below.

- **“Post-pandemic, 30% of U.S. residents plan to take walks more frequently post-COVID than they did before the pandemic, and nearly 15% plan to bike more”**
- **“... those who were frequent walkers or cyclists pre-pandemic expecting more change than those who were not.”**

The calculations for these statements are documented in the Figure 2 data description above.

- **“More than 20% identify taking more walks as an aspect of pandemic life they enjoy.”**

Those survey respondents who responded that there were at least some aspects of pandemic life that they enjoyed were asked to specify up to three, choosing from a list that was generated based on an earlier survey. This tabulation indicates the fraction of the full sample (i.e. including those who do not enjoy any aspects of pandemic life, so representative of the U.S. adult population) that selected “Taking more walks”.

```
. tab enjoy_walks_all [aw=weight_w1b]
```

Enjoy taking more walks	Freq.	Percent	Cum.
Not particularly	5,926.3723	77.85	77.85
Among my top 3 pandemic activities	1,686.6277	22.15	100.00
Total	7,613	100.00	

Urban exodus?

For the analysis of urban vs. non-urban movers, zip codes are classified as dense urban neighborhoods if they have a housing unit density of at least 2000 units per square kilometer. Using this cutoff, about 15% of our weighted sample lived in dense urban neighborhoods. This is somewhat higher than the U.S. as a whole because our sampling strategy oversampled major metropolitan areas. The results reported below of the article are directly calculated from simple tabulations of the survey data, comparing those movers who previously lived in urban neighborhoods to those who previously lived in lower-density neighborhoods.

- **"More than 20% of dense urban employed movers cite not needing to commute as a reason for their move, as opposed to 9% of other employed movers."**

The tabulation below presents the percent of employed movers coming from dense urban areas who reported that a reason for their move was "I do not need to commute", compared to employed movers coming from lower-density neighborhoods.

```
. tab home_move_why_5_w1b urban_pre [aw=weight_w1b] if home_move_why_5_w1b!="Seen
but unanswered" & (worker_pre=="yes" | worker_now=="yes"), col nofreq
```

Moving reason: commute	Moved from neighborhood type		
	Lower-den	Dense urb	Total
I do not need to co..	8.80	21.77	11.23
Not selected	91.20	78.23	88.77
Total	100.00	100.00	100.00

- **"Likewise, 40% of dense urban movers expect to work remotely at least a few times per week post-pandemic, compared to 27% of all other movers."**

```
. tab wfh_exp_comb3 urban_pre [aw=weight_w1b], col nofreq
```

Expected post-pandemic remote work frequency	Moved from neighborhood type Lower-den	Dense urb	Total
Unable	48.58	32.49	45.57
Choose not to	7.05	8.57	7.33
A few times/month	17.24	18.90	17.55
More than once/week	27.14	40.04	29.55
Total	100.00	100.00	100.00

- **"… dense urban movers were not more likely than other movers to be motivated by ei ther pandemic-related public health concerns or by a desire for a more comfortable ho me."**

Parallel to the tabulation above, the following tabulations present the percent of movers coming from urban areas and from lower-density neighborhoods who reported that a reason for their move was "I did not feel safe sharing the house with others", "I did not feel safe in my building or neighborhood due to the virus", and "Moved to a more comfortable home."

```
. tab home_move_why_3_w1b urban_pre [aw=weight_w1b] if home_move_why_3_w1b!="Seen
but unanswered", col nofreq
```

Moving reason: shared home	Moved from neighborhood type Lower-den	Dense urb	Total
I did not feel safe..	7.93	8.29	7.99
Not selected	92.07	91.71	92.01
Total	100.00	100.00	100.00

```
. tab home_move_why_4_w1b urban_pre [aw=weight_w1b] if home_move_why_4_w1b!="Seen
but unanswered", col nofreq
```

Moving reason: virus in neighborhood	Moved from neighborhood type Lower-den	Dense urb	Total
I did not feel safe..	9.63	5.39	8.96
Not selected	90.37	94.61	91.04
Total	100.00	100.00	100.00

```
. tab home_move_why_8_w1b urban_pre [aw=weight_w1b] if home_move_why_8_w1b!="Seen
but unanswered", col nofreq
```

Moving reason: comfortable home	Moved from neighborhood type Lower-den	Dense urb	Total
Moved to a more com..	24.12	26.28	24.46
Not selected	75.88	73.72	75.54
Total	100.00	100.00	100.00
